# Structural Basis of Shiga Toxin Neutralization by Human Antibodies Targeting Canonical Receptor‐Binding and Non‐Canonical Assembly Sites

**DOI:** 10.1002/advs.77402

**Published:** 2026-08-30

**Authors:** Jianting Ke, Xin Li, Xiaoli Wang, Zhaoxia Huang, Shaojie Xue, Yuyang Liu, Jing Pan, Guowei Hu, Yayu Wang, Cheng Wang, Shoudeng Chen

**Affiliations:** ^1^ Division of Nephrology Department of Medicine The Fifth Affiliated Hospital Sun Yat‐sen University Zhuhai Guangdong People's Republic of China; ^2^ Department of Experimental Research The Fifth Affiliated Hospital Sun Yat‐sen University Zhuhai Guangdong People's Republic of China; ^3^ Zhuhai Trinomab Pharmaceutical Co., Ltd Zhuhai Guangdong People's Republic of China; ^4^ NHC Key Laboratory of Clinical Nephrology Sun Yat‐Sen University Guangzhou Guangdong People's Republic of China; ^5^ State Key Laboratory of Anti‐Infective Drug Discovery and Development Sun Yat‐sen University Guangzhou Guangdong People's Republic of China; ^6^ Guangdong Engineering Research Center of Nasopharyngeal Carcinoma Diagnosis and Therapy Guangdong Engineering Technology Research Center of Molecular Imaging the Fifth Affiliated Hospital Sun Yat‐sen University Zhuhai Guangdong People's Republic of China

**Keywords:** Cryo‐EM structure, hemolytic uremic syndrome, human monoclonal antibody, shiga toxin, Stx1aB

## Abstract

Shiga toxin‐producing *Escherichia coli* (STEC) infections cause severe systemic complications, yet targeted antitoxin therapeutics remain an unmet clinical need. Here, we isolated a panel of human monoclonal antibodies (mAbs) targeted at STEC toxin and characterized two potent neutralizing candidates, RDS045 and RDS059. The cryo‐electron microscopy structure of the human mAb RDS059 in complex with the Shiga toxin 1a B subunit (Stx1aB) reveals that RDS059 recognizes the surface‐exposed loop of Stx1aB, whereby it directly competes with the host glycolipid globotriaosylceramide (Gb3) to block toxin‐cell engagement. In contrast, RDS045 binds a non‐canonical quaternary epitope at the pentameric interface of Stx1aB, acting as an interprotomer clamp via an extensive hydrogen‐bonding and cation‐π interaction network. Both antibodies confer robust protection in cellular assays and an in vivo murine Stx1a challenge model. Collectively, this work defines two distinct molecular blueprints for Shiga toxin receptor blockade and assembly interference, establishing mechanistic frameworks for the development of STEC therapeutics.

## Graphical abstract

Cryo‐electron microscopy structures reveal two distinct neutralization mechanisms of fully human monoclonal antibodies targeting the Shiga toxin 1a B subunit. RDS059 occludes the Gb3 receptor‐binding site, whereas RDS045 engages a lateral quaternary epitope to disrupt holotoxin assembly. Both antibodies exert potent neutralizing activity and confer robust protection in murine toxin challenge models.

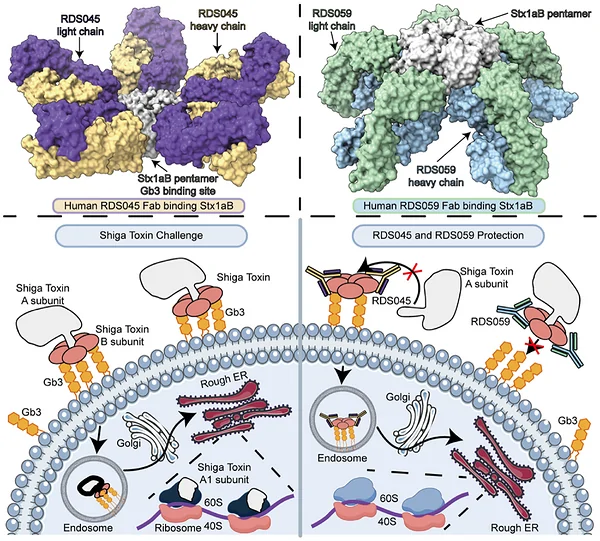

## 1 Introduction

Shiga toxin (Stx) is a potent ribosome‐inactivating exotoxin produced by pathogenic Shigella species and multiple pathogenic *Escherichia coli* strains, functioning to potently inhibit host cellular protein synthesis [1]. Since the discovery of the dysentery bacillus by Kiyoshi Shiga in 1898, Stx has been recognized as the pivotal etiological driver of diarrhea and dysentery [2, 3]. Pathogenic *E. coli* strains capable of producing Stx are classified as Shiga toxin‐producing *E. coli* (STEC) owing to their consistent induction of hemorrhagic intestinal pathologies [4, 5]. Stx family toxins are categorized into two major types, Stx1 and Stx2, and share an A‐B_5_ heterohexameric architecture [4, 6]. The enzymatic A subunit (approximately 32 kDa) mediates cytotoxicity by inactivating ribosomes, while the pentameric B subunit (approximately 8 kDa per monomer) facilitates recognition of the host cell surface glycolipid globotriaosylceramide (Gb3) and mediates host cell attachment [7, 8, 9].

Systemic dissemination of Shiga toxin from the intestines to the bloodstream can lead to life‐threatening complications, including renal failure due to acute cortical necrosis, hemorrhagic colitis (HC), hemolytic uremic syndrome (HUS), and central nervous system (CNS) dysfunction [10, 11, 12]. Clinically, HUS is defined by the triad of microangiopathic hemolytic anemia, thrombocytopenia, and acute renal failure. An estimated 90% of HUS cases are triggered by Stx‐producing bacteria and are classified as typical HUS [13]. The remaining 5%–10% are classified as atypical HUS (aHUS), which is primarily driven by dysregulation of the complement system and is frequently associated with mutations in complement regulatory genes [14].

In contrast to aHUS, for which complement‐directed therapy has provided a disease‐targeted treatment strategy [15], no specific therapeutics are approved for STEC infection or typical HUS. Some clinical reports suggest that certain antibiotics, such as sulfonamides or β‐lactams, may paradoxically increase the risk of developing HUS [16, 17, 18, 19]. Proposed mechanisms include antibiotic‐induced bacterial lysis leading to heightened toxin release, or alterations in gut microbiota dynamics that favor STEC colonization and enhance toxin absorption [20, 21, 22]. Furthermore, antibiotic treatment may trigger the bacterial SOS response, potentially upregulating Shiga toxin production [10, 19].

To address this unmet clinical demand, multiple antibody‐based antitoxin candidates have been developed for STEC‐associated HUS. However, therapeutic development for STEC‐associated HUS has remained limited. Among antibody‐based strategies evaluated clinically, direct anti‐Stx programs include Shigamabs (cαSTX1 and cαSTX2) [23], Urtoxazumab (TMA‐15) [24], and INM‐004 [25]. Complement blockade with Eculizumab, originally developed for aHUS, has also been explored in this setting [26]. The development of Shigamabs and Urtoxazumab has been discontinued. INM‐004, a broad‐spectrum neutralizing equine F(ab')_2_ antibody developed by Inmunova SA, is currently in Phase III clinical trials in Argentina (NCT06389474).

Building on our previously reported technology [27, 28], we isolated a panel of human antibodies from sorted single B cells from human donors via fluorescence‐activated cell sorting (FACS). We demonstrated that two of the isolated monoclonal antibodies (mAbs), RDS045 and RDS059, exert significant protective effects against Stx in both in vitro and in vivo assays. Moreover, we utilized high‐resolution single‐particle cryo‐electron microscopy (cryo‐EM) to elucidate the structural basis of the mAbs' interaction with the toxin pentameric B subunits. Our results reveal that RDS059 directly outcompetes Gb3 for binding to the toxin's cognate receptor. Conversely, RDS045 engages a previously unrecognized lateral epitope on the B pentamer. This lateral binding functions as an interprotomer molecular clamp, exerting a disruptive effect on the assembly and structural stability of the holotoxin. By defining these two distinct blueprints for neutralization, our work expands the structural landscape of Stx inhibition and provides a framework for developing next‐generation, mechanism‐diverse therapeutics.

## 2 Results

### 2.1 Serological Screening and Single‐B‐Cell Cloning Identify Two Lead Stx1aB‐Neutralizing Antibodies

**FIGURE 1:**
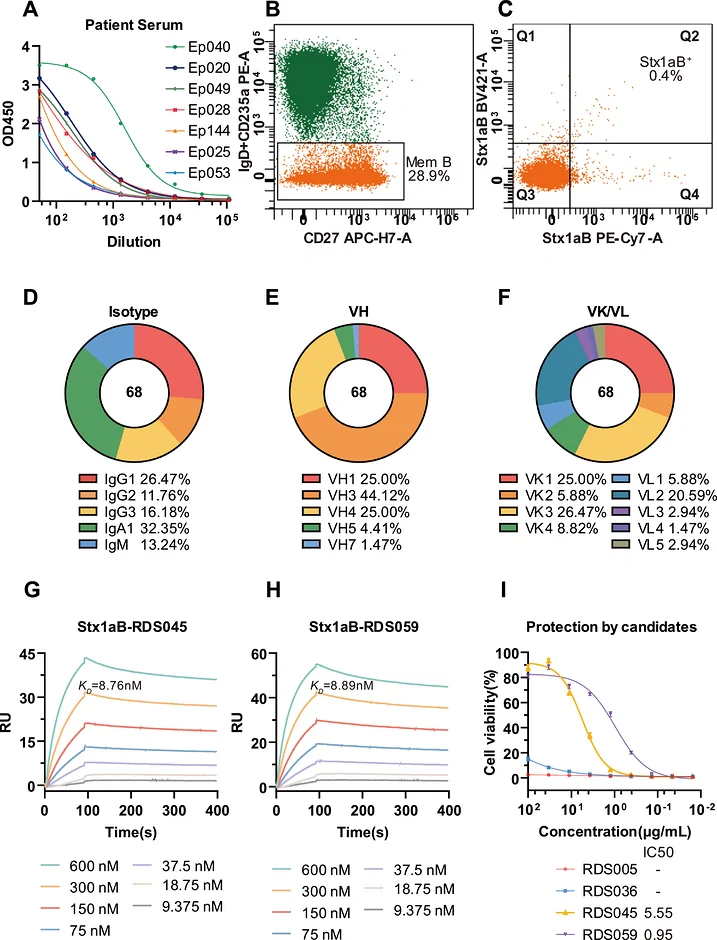
Isolation and Functional Characterization of Fully Human Neutralizing Antibodies Targeting Stx1aB. (A) Serum reactivity against rStx1aB in patients with renal abnormalities, measured by ELISA. Serial dilutions of patient sera were tested, and binding was detected as OD450. (B) Representative flow cytometry plot showing identification of memory B cells from peripheral blood mononuclear cells. Memory B cells were defined as IgD^−^ CD235a^−^ CD27^+^ cells. (C) Representative flow cytometry plot showing staining of antigen‐specific memory B cells with fluorescently labeled Stx1aB. Stx1Ab^+^ B cells were sorted as single cells for antibody gene amplification and recombinant monoclonal antibody expression. (D) Isotype distribution of the 68 recovered Stx1aB‐reactive monoclonal antibodies. (E) Variable heavy chain (VH) gene family usage among the 68 Stx1aB‐reactive monoclonal antibodies. (F) Variable light‐chain gene family usage among the 68 Stx1aB‐reactive monoclonal antibodies. (G) Binding kinetics of RDS045 to rStx1aB measured by SPR. Representative sensorgrams are shown, with a calculated equilibrium dissociation constant (K_D_) of 8.76 nm. (H) Binding kinetics of RDS059 to rStx1aB Stx1aB measured by SPR. Representative sensorgrams are shown, with a calculated equilibrium dissociation constant (K_D_) of 8.89 nm. (I) Neutralization of Stx1a‐induced cytotoxicity in VeroE6 cells by candidate monoclonal antibodies. Cell viability was measured after incubation with Stx1a in the presence of the indicated antibodies. RDS045 and RDS059 showed potent neutralizing activity, whereas RDS005 and RDS036 did not. IC50 values are indicated. Data are representative of at least 3 independent experiments or shown as mean ± SEM, as indicated. Statistical analyses are described in STAR Methods.

To identify human monoclonal antibodies targeting the B subunit of Shiga toxin 1a (Stx1aB), we first screened sera from hospitalized patients with renal abnormalities, including proteinuria and hematuria, for reactivity against recombinant Stx1aB (rStx1aB) via enzyme‐linked immunosorbent assay (ELISA). Among seven tested donors, Ep040 exhibited the strongest antigen‐binding signal across serial dilutions, whereas the other samples displayed substantially weaker reactivity (Figure 1A). Given this robust seroreactivity, Ep040 was selected for downstream antigen‐specific single‐B‐cell screening.

Peripheral blood mononuclear cells (PBMCs) isolated from donor Ep040 were subjected to multicolor flow cytometric sorting to isolate Stx1aB‐reactive memory B cells. After sequential gating on singlets and CD3^−^CD14^−^CD16^−^CD20^+^ B cells, the memory B cell compartment was defined as CD27^+^IgD^−^/low cells (Figure 1B; Figure S1A). Within this population, Stx1aB‐reactive cells were identified using dual fluorescently labeled Stx1aB probes, with PE‐Cy7‐ and BV421‐conjugated Stx1aB used in parallel to increase the specificity of antigen capture (Figure 1C). Cell counts and frequencies at individual gating steps were summarized in Figure S1B. The variable regions of immunoglobulin (Ig) heavy and light chain gene segments (VH and VK/VL, respectively) from sorted dual‐positive single memory B cells were amplified by reverse transcription‐polymerase chain reaction (RT‐PCR), followed by sequencing, germline annotation, and recombinant mAb expression using previously described methods [27, 28].

Among the 68 isolated antigen‐reactive pairs, IgA1 and IgG1 isotypes were identified as the predominant subsets, accounting for 32.35% and 26.47% of the total antibody repertoire, respectively (Figure 1D). Profiling of the VH gene repertoire showed that 44.12% of heavy chain variable genes were derived from the VH3 family, with 25% originating from the VH4 family (Figure 1E). In contrast to restricted VH gene usage, light chain variable gene clusters displayed substantially higher diversity. VK1 encoded 25% of the total antibodies, VK3 contributed 26.47%, and VL2 accounted for 20.59% of the repertoire (Figure 1F). The extent of divergence from the germline in the heavy and light chains, as well as the distribution of divergence from germline lengths, were shown in Figure S2A,B.

To identify functional candidates, all 68 recombinant antibodies were expressed and screened for Stx1aB binding by ELISA. While most recombinant antibodies showed little or undetectable binding in the initial screen, four clones were identified as Stx1aB‐reactive candidates, each of which exhibited concentration‐dependent binding to Stx1aB by ELISA. The apparent EC50 values were 0.0045 µg/mL for RDS005, 0.0037 µg/mL for RDS036, 0.0032 µg/mL for RDS045, and 0.0064 µg/mL for RDS059 (Figure S2C,D). Sequence analysis indicated that the four Stx1aB‐reactive antibodies were genetically distinct, using three IGHV genes from two IGHV families together with different D/JH and light‐chain gene combinations, consistent with their derivation from independent B‐cell lineages (Table S1). These results established four human Stx1aB‐reactive monoclonal antibodies for subsequent analyses.

We next performed surface plasmon resonance (SPR) to quantify the binding kinetics and affinity of all four candidate mAbs. All four antibodies bound Stx1aB with nanomolar‐range affinities (Figure 1G,H, Figure S2E,F). Kinetic fitting yielded equilibrium dissociation constants (K_D_) of 34.8 nm for RDS005, 7.43 nm for RDS036, 8.76 nm for RDS045, and 8.89 nm for RDS059 (Figure 1G,H, Figure S3A,B and Table S2). We further assessed whether differential binding affinity correlated with toxin neutralization potency. We established 1 µg/mL Shiga toxin 1a (Stx1a) holotoxin as the working concentration for subsequent testing in a VeroE6 cell‐based protection assay (Figure S2G). Under these conditions, only RDS045 and RDS059 conferred dose‐dependent protection against Stx1a cytotoxicity, whereas RDS005 and RDS036 showed no measurable protective effect (Figure 1I). Taken together, these functional assays prioritized two lead neutralizing mAbs, RDS045 and RDS059, for subsequent structural and mechanistic investigation.

### 2.2 Distinct Modes of mAbs Neutralizing Engagement on the Stx1aB Pentamer

To define how the two protective antibodies engage Stx1aB, purified rStx1aB was incubated with Fab fragments of RDS045 or RDS059, and the resulting antigen‐antibody complexes were isolated by size‐exclusion chromatography for cryo‐EM analysis (Figures S3 and S4). Cryo‐EM reconstruction yielded high‐resolution maps for both complexes, with overall resolutions of 2.00 Å for the RDS059‐rStx1aB pentamer and 2.12 Å for the RDS045‐rStx1aB pentamer. Complete statistics for data collection, processing, and refinement are summarized in Table S3.

**FIGURE 2:**
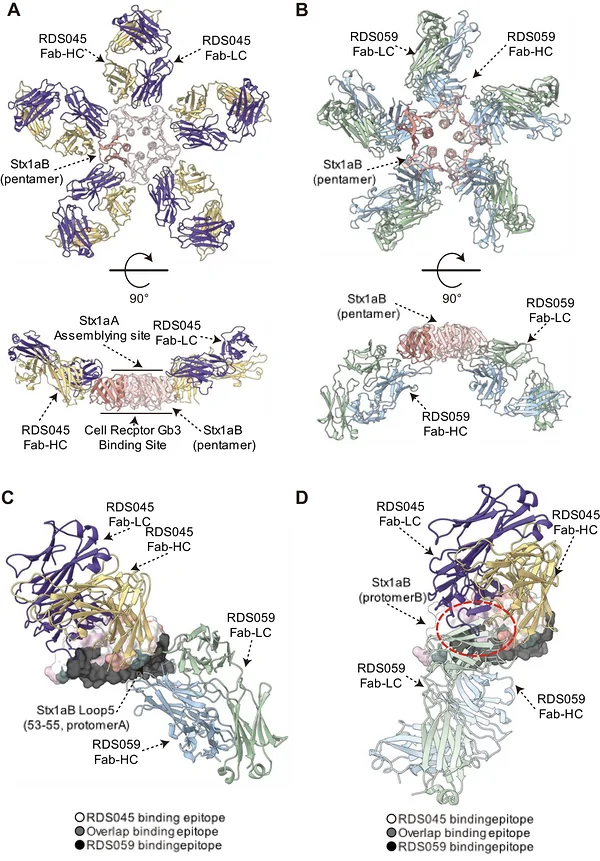
Cryo‐EM Structures Reveal Distinct Binding Topologies of RDS045 and RDS059 on the Stx1aB Pentamer. (A) Cryo‐EM structure of the Stx1aB pentamer in complex with RDS045 Fab shown in top and side views. The Stx1aB pentamer is shown in pink, with the heavy and light chains of RDS045 Fab shown in gold and purple, respectively. RDS045 binds the lateral surface of the Stx1aB pentamer, spatially separated from the Gb3 receptor‐binding site and proximal to the Stx1aA assembly‐facing region. (B) Cryo‐EM structure of the Stx1aB pentamer in complex with RDS059 Fab shown in top and side views. The Stx1aB pentamer is shown in pink, with the heavy and light chains of RDS059 Fab shown in blue and green, respectively. RDS059 binds the membrane‐distal, receptor‐facing surface of the Stx1aB pentamer in a position proximal to the Gb3‐binding region. (C, D) Superposition of the RDS045–Stx1aB and RDS059–Stx1aB complexes. The Stx1aB protomer A is shown in pink, protomer B in magenta.RDS045 Fab is shown in purple and yellow, and RDS059 Fab in light green and blue, with the corresponding heavy‐ and light‐chain regions indicated. An overlapping epitope is observed at the peripheral boundary of residues 53–55. The epitope regions are indicated according to the color scheme shown below the panel.

Both antibodies bound the Stx1aB pentamer but adopted strikingly different binding topologies. As shown in Figure 2A, RDS045 recognized a non‐canonical quaternary epitope spanning two adjacent protomers of the Stx1aB pentamer in the top‐down view, with its antigen‐binding interface jointly contributed by residues from neighboring B subunits. The side view of the complex further revealed that RDS045 bound exclusively opposite to the Gb3 receptor‐binding surface of the pentamer and was oriented directly toward the Stx1aA assembly interface. This binding position placed RDS045 across the interprotomer gaps of the B pentamer, indicating that its recognition depends on the intact pentameric quaternary structure and that RDS059 cannot bind to individual dissociated B subunits.

By contrast, RDS059 engaged each Stx1aB protomer individually on the apical, Gb3 receptor‐binding surface of the pentamer (Figure 2B). In the top view, five RDS059 Fab molecules symmetrically decorated the receptor‐facing face of the Stx1aB pentamer at a 1:1 Fab‐to‐protomer stoichiometry, with each Fab contacting only one B subunit rather than bridging adjacent protomers. The side view confirmed that the entire binding footprint of RDS059 overlaps with the known Gb3‐binding region on Stx1aB, consistent with a receptor‐competing neutralization mechanism.

Superposition of the RDS045‐Stx1aB and RDS059‐Stx1aB complexes, based on alignment of the Stx1aB pentamer core, showed that the two antibodies recognize largely distinct surfaces of the B pentamer (Figure 2C). RDS045 binds primarily to the lateral interprotomer surface, whereas RDS059 engages the basal surface of the B subunit. Residue‐level mapping identified a small epitope overlap involving Stx1aB residues 53–55. In addition to this epitope overlap, we modeled RDS059 binding to protomer B and examined its spatial relationship with RDS045. As shown in Figure 2D, the light chain of RDS059 substantially overlaps with the bound RDS045 Fab, indicating steric interference between the two antibodies. Thus, the structural arrangement would not support simultaneous binding of five RDS045 Fabs and five RDS059 Fabs to a single Stx1aB pentamer.

To determine whether RDS045 and RDS059 compete for binding to Stx1aB, we performed an in‐tandem BLI competition assay using biotinylated recombinant Stx1aB immobilized on streptavidin biosensors (Figure S5). Pre‐binding of RDS045 inhibited subsequent RDS059 binding by approximately 40.7% (Figure S5A), whereas pre‐binding of RDS059 inhibited subsequent RDS045 binding by approximately 72.3% (Figure S5B). The two antibodies therefore showed incomplete competition, with RDS059 more strongly inhibiting RDS045 binding. These results are consistent with the epitope overlap and steric interference observed in the structural analysis.

Structure‐guided complementarity‐determining region (CDR) assignment, together with IMGT‐standard sequence annotation of the VH and VL domains, provided residue‐level context for the distinct paratopes underlying these two binding modes (Figure S6). The paratope of RDS045 engaged residues from multiple CDR loops to match the extended, interprotomer quaternary epitope, whereas the paratope of RDS059 was configured to recognize the compact, surface‐exposed receptor‐binding loop on a single protomer.

Collectively, these structural observations demonstrated that the two protective antibodies targeted Stx1aB through fundamentally different molecular recognition modes, suggesting they neutralized Stx1a through mechanistically distinct routes.

### 2.3 RDS059 Neutralizes Toxin by Occluding the Receptor‐Binding Face in a Loop5 Centered Manner

**FIGURE 3:**
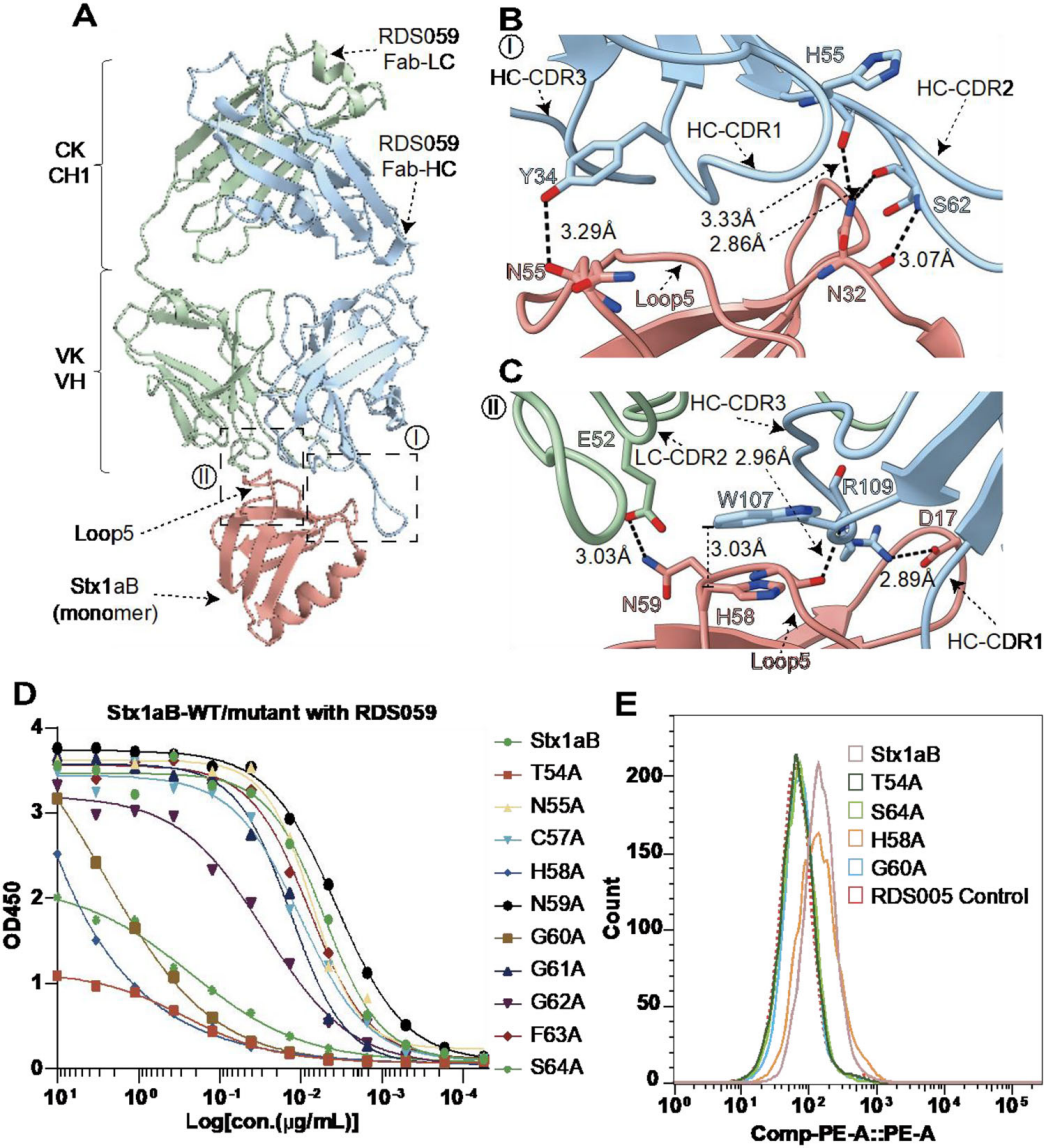
Structural and Functional Analyses Define loop5‐centered Recognition of Stx1aB by RDS059. (A) Overall structure of RDS059 Fab bound to a Stx1aB monomer. The heavy and light chains of RDS059 Fab are shown in blue and green, respectively, and Stx1aB is shown in pink. The boxed regions indicate the local interfaces enlarged in (B) and (C). (B) Close‐up view of interface I, showing contacts between RDS059 heavy‐chain CDRs and Stx1aB loop 5. Hydrogen bonds and polar contacts are indicated by dashed lines, with distances shown in angstroms. (C) Close‐up view of interface II, showing additional interactions formed by RDS059 heavy‐ and light‐chain CDRs with Stx1aB residues surrounding loop 5. Hydrogen bonds and polar contacts are indicated by dashed lines, with distances shown in angstroms. (D) Binding of RDS059 to wild‐type Stx1aB and alanine‐substituted Stx1aB variants, measured by ELISA. Substitutions at residues within the mapped epitope differentially affected RDS059 binding, identifying key contact residues on Stx1aB. (E) Flow cytometry analysis of selected Stx1aB alanine variants detected with the control antibody RDS005. Comparable staining profiles for most variants indicated that the reduced RDS059 binding observed for selected mutants was not primarily due to global loss of antigen integrity.

Having established the overall cryo‐EM structures of the mAb‐Stx1aB complexes at high resolution, we next performed atom‐level dissection of the antigen‐antibody interfaces to delineate the specific molecular interactions that underpin the distinct neutralizing mechanisms of these two antibodies. For the RDS059‐Stx1aB complex, structural analysis at the monomeric level revealed that all direct antigen contacts were mediated exclusively by the variable domains of the heavy and light chains (VH and VK). Further epitope mapping localized the RDS059 binding site to a contiguous surface region centered on the loop5 motif of Stx1aB, with two discrete contact clusters identifiable within the interface that were displayed in the magnified inset views (Figure 3A).

Detailed inspection of the interface revealed an extensive network of polar contacts that stabilized the complex, involving all three complementarity‐determining regions of the heavy chain (HC‐CDR1, HC‐CDR2 and HC‐CDR3) as well as CDR2 of the light chain. Multiple hydrogen bonds were formed between the antibody CDR loops and residues within and immediately flanking loop5 of Stx1aB. In the first contact cluster, the side chain of Tyr34 from HC‐CDR1 formed a hydrogen bond with the side chain of Asn55 on Stx1aB at a distance of 3.29 Å. Additional backbone and side‐chain hydrogen bonds with measured lengths of 2.86 and 3.33 Å bridged HC‐CDR1 and HC‐CDR3 to loop5‐adjacent residues. Ser62 from HC‐CDR2 also contributed a hydrogen bond to Asn32 of Stx1aB with a distance of 3.07 Å (Figure 3B). This multivalent array of polar interactions anchored the Fab fragment firmly to the receptor‐facing surface of the B subunit.

In the second contact cluster, a prominent aromatic stacking interaction formed between Trp107 located in HC‐CDR3 and His58 within loop5 of Stx1aB. This *π–π* stacking interaction provided strong hydrophobic and aromatic stabilization to the interface and represents a core energetic contributor to binding affinity. Surrounding this central aromatic pair, additional hydrogen bonds further reinforced the interaction network. Glu52 from light‐chain CDR2 formed a 3.03 Å hydrogen bond with Asn59 of Stx1aB, and Asp17 from HC‐CDR1 formed a 2.89 Å contact with residues in the immediate vicinity of His58 (Figure 3C). The combination of polar hydrogen bonding and aromatic stacking created a well‐defined, high‐affinity paratope that matched the conformational features of the loop5‐centered epitope.

To determine whether this binding mode could interfere with receptor recognition, we superposed the RDS059‐Stx1aB complex onto the Gb3‐bound Stx1aB structure. This analysis showed that the RDS059 paratope sterically encroached on the receptor‐binding face of Stx1aB, with heavy‐chain CDR1‐3 projecting into the space occupied by the three Gb3 analogues (Figure S7A,B).

These observations supported a model in which RDS059 broadly interferes with productive toxin‐receptor engagement across the receptor‐binding surface, rather than occluding only a single receptor‐binding site. Guided by this structural model, we performed alanine‐scanning mutagenesis to assess the contribution of loop5 residues to RDS059 binding. Substitution of T54, H58, G60, or S64 with alanine reduced RDS059 binding in ELISA (Figure 3D).

We then asked whether these residues also contributed to RDS059‐mediated blockade of Stx1aB binding to VeroE6 cells. Flow cytometric analysis showed that the H58A substitution caused clear loss of RDS059‐mediated competition, whereas the other loop5 mutants remained suppressible by the antibody (Figure 3E, Figure S7C).

Together, the structural and functional data supported a unified model for RDS059‐mediated neutralization. The antibody engaged a loop5‐centered conformational epitope on the receptor‐binding face of Stx1aB through a combination of extensive hydrogen bonding networks and a critical aromatic stacking interaction mediated by His58. This binding event positioned the antibody variable domains to exert broad steric hindrance across the Gb3‐binding surface, thereby effectively preventing toxin attachment to host cell surfaces and neutralizing Stx1a cytotoxicity.

### 2.4 Monoclonal Antibody RDS045 Recognizes a Non‐Canonical Lateral Epitope on Stx1aB Pentamer

Structural analysis of the cryo‐EM reconstruction showed that RDS045 binds a non‐canonical epitope on the lateral surface of the Stx1aB pentamer rather than the canonical Gb3‐binding face (Figure 2A). In a 90° rotated view, the antibody footprint was positioned on the outer sidewall of the pentamer and did not overlap with previously defined Gb3‐binding residues (Figure 2A, Figure S7D), indicating that RDS045 was unlikely to neutralize Stx1a through direct occlusion of the receptor‐binding pockets. This topology differed from that of previously described receptor‐blocking anti‐Stx nanobodies, including Nb113 (targeting Stx2aB) and NbStx2e1 (targeting Stx2eB), both of which engaged the receptor‐facing surface of the B5 ring (Figure S7E).

**FIGURE 4:**
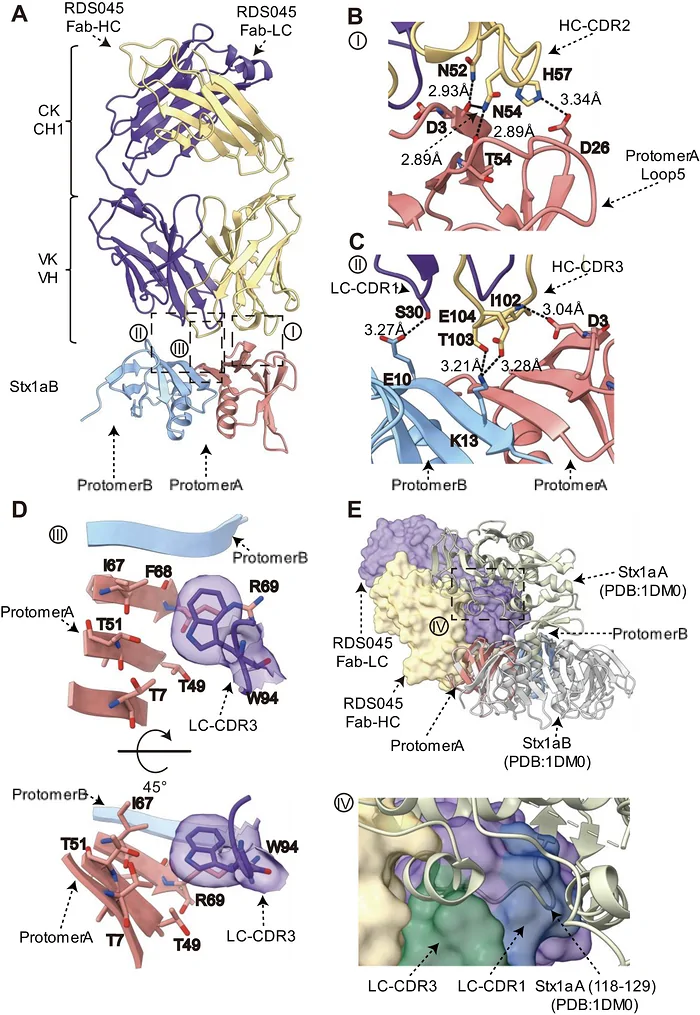
Structural analysis reveals quaternary lateral recognition of the Stx1aB pentamer by RDS045. (A) Overall structure of RDS045 Fab bound to the lateral surface of the Stx1aB pentamer. The heavy and light chains of RDS045 Fab are shown in gold and purple, respectively. Two adjacent Stx1aB protomers that contribute to the epitope are shown as protomer A (pink) and protomer B (blue). The boxed regions indicate the local interfaces enlarged in (B–D). (B) Close‐up view of interface I, showing contacts between RDS045 heavy‐chain CDRs and protomer A. Hydrogen bonds and polar contacts are indicated by dashed lines, with distances shown in angstroms. (C) Close‐up view of interface II, showing a cross‐protomer interaction network formed by HC‐CDR3 and LC‐CDR1 of RDS045 with residues from protomers A and B. Hydrogen bonds and polar contacts are indicated by dashed lines, with distances shown in angstroms. (D) Close‐up view of interface III, showing insertion of LC‐CDR3 residue W94 into a lateral pocket on protomer A. Two views are shown. W94 forms a cation‐pi interaction with R69 and is surrounded by hydrophobic residues contributed by the lateral surface of protomer A. (E) Superposition of the RDS045‐bound Stx1aB structure with the holotoxin structure of Stx1a (PDB: 1DM0). Upper panel, overall view showing the position of RDS045 relative to Stx1aA and the B‐subunit pentamer. Lower panel, enlarged view showing proximity of LC‐CDR1 and LC‐CDR3 to the Stx1aA region spanning residues 118–129.

We next examined the local antibody‐antigen interface in detail. RDS045 recognized a composite quaternary epitope spanning two adjacent Stx1aB protomers, designated protomers A and B, thereby forming an inter‐protomer clamp on the lateral surface of the assembled pentamer (Figure 4A). The interface was stabilized by an extensive hydrogen‐bond network distributed across both protomers. Residues N52, N54, and H57 of heavy chain CDR2 contacted D3, D26, and T54 of protomer A (Figure 4B). In addition, heavy chain CDR3 together with light chain CDR1 bridged the interface between protomers A and B, forming a cross‐protomer hydrogen‐bond network involving E10 and K13 of protomer B and D3 of protomer A (Figure 4C). These interactions defined a quaternary epitope that is presented only in the context of the assembled B‐subunit pentamer. The interface was further reinforced by W94 from light chain CDR3, which wedged into a hydrophobic pocket on protomer A formed by T7, T49, T51, I67, F68, and R69 (Figure 4D). In this configuration, the indole ring of W94 formed a cation‐π interaction with R69, while its NE1 atom was oriented toward T51 OG1, further contributing to stabilization of the lateral binding mode.

Superposition with the holotoxin structure further placed light chain CDR1 and light chain CDR3 in close proximity to the Stx1aA region spanning residues 118–129 (Figure 4E), suggesting that lateral engagement by RDS045 may impose steric constraints on holotoxin architecture. Comparison of the RDS045‐bound Stx1aB pentamer with the holotoxin‐associated Stx1aB pentamer (PDB: 1DM0) yielded a core RMSD of 0.829 Å across 345 aligned residues, indicating that the overall pentameric architecture was largely preserved (Figure S8A). B‐factor surface mapping showed relatively lower B factors for chain E than for the other protomers in the holotoxin‐associated structure, whereas the five protomers in the RDS045‐bound structure displayed a more uniform B‐factor distribution (Figure S8B,C). To further characterize these structural differences, we performed residue‐wise Cα positional‐deviation and normalized B‐factor analyses. Because the A subunit was positioned at the interface between chains D and E, these two protomers were analyzed as a subgroup. Their mean Cα positional deviations were generally smaller than those of the other three protomers (Figure S8D). The normalized B‐factor analysis showed lower relative values in the basal portion of α1 near the Gb3‐binding surface and higher relative values in its C‐terminal region in the RDS045‐bound structure (Figure S8E).

Collectively, these findings indicate that RDS045 binding does not induce a substantial global rearrangement of the Stx1aB pentamer but is associated with localized positional and relative B‐factor differences. This structural comparison is consistent with the neutralization mechanism proposed above, whereby lateral engagement of RDS045 sterically interferes with A‐subunit association while preserving the overall B‐pentamer architecture.

Conservation mapping across 19 Stx subtypes further showed that the RDS045 footprint localizes to a relatively conserved lateral patch on the B subunit, while the RDS059 footprint mapped to a distinct receptor‐facing region with a different conservation pattern (Figure S9A,B). The sequence alignment further showed that most RDS045‐contacting residues were conserved or conservatively substituted among the Stx1 subtypes, whereas several corresponding positions differed in the more divergent Stx2 subtypes (Figure S9C). The RDS059 epitope also included conserved residues, but its conserved and variable positions were distributed differently across the epitope (Figure S9B,C). Together, these findings identify RDS045 as targeting a relatively conserved lateral epitope on the Stx1aB pentamer that is structurally distinct from the canonical receptor‐binding face and is consistent with a neutralization mode that does not rely on direct competition with Gb3.

### 2.5 Therapeutic Activity of RDS045 and RDS059 in a Murine Stx1a Challenge Model

**FIGURE 5:**
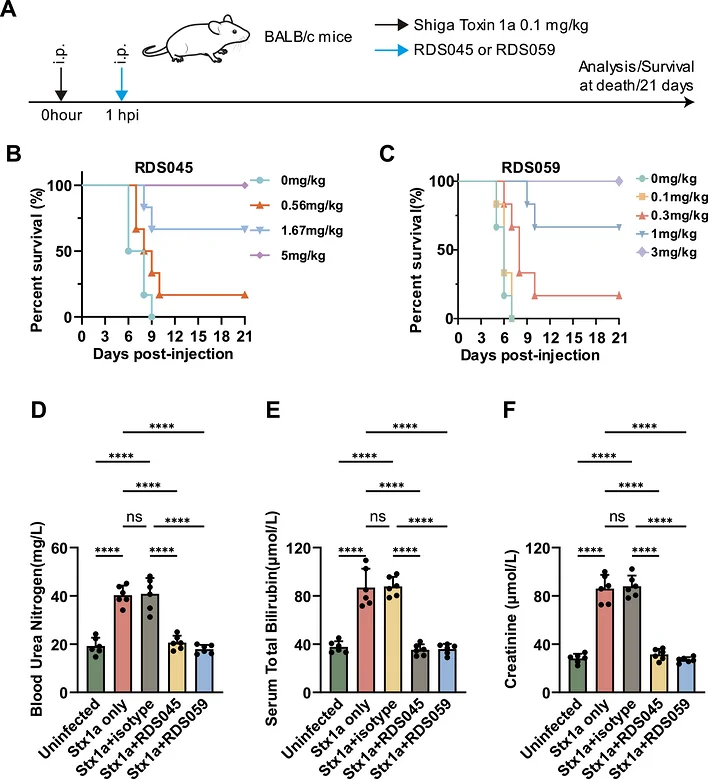
RDS045 and RDS059 confer post‐exposure protection in a murine Stx1a holotoxin challenge model. (A) Schematic of the experimental design. BALB/c mice were challenged intraperitoneally with Stx1a holotoxin on day 0 and treated 1 h later with the indicated antibodies. (B and C) Kaplan‐Meier survival curves of Stx1a holotoxin‐challenged mice treated with the indicated doses of RDS045 (B) or RDS059 (C) (n = 6 per group). Survival was analyzed using the log‐rank (Mantel‐Cox) test. Overall survival differed significantly among groups (P<0.001). A significant dose‐dependent trend was also observed (P<0.0001). (D‐F) Blood urea nitrogen (BUN) (D), serum total bilirubin (E), and serum creatinine (F) levels in unchallenged mice, Stx1a holotoxin‐challenged mice without antibody treatment, Stx1a holotoxin‐challenged mice treated with isotype control antibody, and Stx1a holotoxin‐challenged mice treated with RDS045 (5 mg/kg) or RDS059 (3 mg/kg) (n = 6 per group). Data are shown as mean ± SD. Biochemical data were analyzed by one‐way ANOVA followed by Dunnett's multiple‐comparisons test using the Stx1aA only or Stx1a + isotype group as the reference group. ^*^ *p* < 0.05, ^**^ *p* < 0.01, ^***^ *p* < 0.001, ^****^ *p* < 0.0001.

To determine whether the distinct structural neutralization mechanisms of RDS045 and RDS059 translated into protective efficacy in vivo, we evaluated both antibodies in a lethal murine Stx1a holotoxin challenge model. BALB/c mice aged 4 to 6 weeks were randomly assigned to experimental groups, with six animals included in each group. All mice received an intraperitoneal injection of Stx1a holotoxin on day 0, and a single dose of the test antibody was administered via the same route 1 h after toxin challenge. Mice were monitored daily for 21 days to track survival status, body weight changes, and clinical signs of intoxication, with the full experimental workflow summarized in Figure 5A.

Mice in the Stx1a‐only and Stx1a plus antibodies isotype control groups developed evident disease manifestations starting at 3 to 5 days after injection. Most mice in these two control groups succumbed to toxin‐induced injury within 9 days of challenge, verifying the lethality and stability of the selected toxin dose. Both RDS045 and RDS059 conferred dose‐dependent survival protection against lethal Stx1a intoxication. For RDS045, the 0.56 mg/kg dose provided only limited survival benefit, with a mild prolongation of median survival time but eventual mortality in most animals. At 1.67 mg/kg, RDS045 delivered substantial partial protection, with approximately 60% of mice surviving to the 21‐day observational endpoint. The 5 mg/kg dose achieved complete and durable protection, as all mice in this group survived through the entire 21‐day observation period without severe signs of intoxication (Figure 5B).

RDS059 showed a comparable dose‐dependent protective profile, with slightly higher overall potency relative to RDS045. Doses of 0.1 and 0.3 mg/kg produced limited survival benefit, with only modest extensions of median survival time relative to the control group. The 1 mg/kg dose conferred partial protection, with roughly 60% of mice remaining alive at the end of the 21‐day observation window. Complete survival protection was achieved at the 3 mg/kg dose, at which all mice survived the full study period with minimal clinical signs of toxicity (Figure 5C). These data confirmed that both antibodies retained robust in vivo therapeutic efficacy when delivered after toxin exposure, despite operating through fundamentally different neutralization mechanisms.

We next assessed whether antibody treatment could alleviate biochemical markers of toxin‐associated tissue injury. Blood samples were collected at the experimental endpoint or at the time of moribund euthanasia for measurement of circulating biomarkers of renal and systemic function. Compared with uninfected control mice, Stx1a holotoxin challenge caused pronounced and statistically significant elevations in blood urea nitrogen, serum creatinine and serum total bilirubin, consistent with severe acute kidney injury and systemic tissue damage. The isotype control antibody did not confer any detectable protective effect, as all three biochemical parameters in the isotype treatment group were statistically indistinguishable from those in the toxin‐only group (Figure 5D–F). By contrast, treatment with either RDS045 or RDS059 substantially reversed all toxin‐induced biochemical abnormalities. Both antibodies reduced blood urea nitrogen and serum creatinine to levels close to those of uninfected animals, with statistically significant differences compared with the isotype control group. These results indicated that both antibodies effectively mitigate Stx1a‐induced acute renal dysfunction. In parallel, serum total bilirubin levels were also significantly lowered by antibody treatment, reflecting improvement of toxin‐associated systemic and hepatic injury. No statistically significant differences were observed between the RDS045 and RDS059 treatment groups for any of the measured biomarkers, indicating equivalent organ‐protective activity for the two antibodies at their fully protective doses.

Together, these in vivo data demonstrated that both antibodies ameliorated Stx1a‐induced renal injury and systemic biochemical damage in mice, despite acting through distinct structural mechanisms. The dose‐dependent survival benefit and organ‐protective effects observed for both candidates provided robust preclinical evidence to support their further development as targeted therapeutics for STEC‐associated disease.

## 3 Discussion

Stx1a‐mediated hemolytic uremic syndrome (HUS) and systemic toxicity lack approved targeted therapeutics, making neutralizing antibodies a critical research focus for STEC‐associated disease intervention. Our study confirms that human anti‐Stx1aB monoclonal antibodies RDS045 and RDS059 exert potent neutralizing activity against Stx1a in both the in vitro VeroE6 cell model and in vivo murine Stx1a holotoxin challenge model, with robust therapeutic efficacy in lethal toxin challenge assays. These findings provide direct experimental support for Stx1a neutralizing antibodies as candidate therapeutics for Stx‐mediated disease, an area of substantial unmet need in which antibiotics remain problematic and anti‐toxin biologics are still limited, with the equine F(ab')_2_ product INM‐004 representing one of the few clinically advanced programs [25]. Beyond demonstrating efficacy, our data further indicate that protective antibodies against Stx1aB are not mechanistically uniform. Rather, the two antibodies identified here recognize the same toxin subunit through topologically distinct binding modes, suggesting that the Stx1aB pentamer contains more than one exploitable neutralizing surface for therapeutic targeting.

One of these modes is represented by RDS059, which binds the receptor‐proximal face of the Stx1aB pentamer in a configuration positioned to interfere directly with host receptor engagement. In this respect, RDS059 is conceptually closer to previously described anti‐Stx nanobodies such as Nb113 [29] and NbStx2e1 [30], both of which were derived from camelid immunization or llama VHH platforms and recognize the receptor‐facing surface of Stx B‐subunit assemblies. Structural studies of those nanobodies support a neutralization logic dominated by steric competition with glycolipid receptor binding. RDS059 therefore provides an important internal point of reference in our study. It confirms that the canonical receptor‐facing surface remains a productive vulnerability on Stx1aB, while also extending this mechanism into the context of fully human monoclonal antibody discovery. This distinction is therapeutically relevant because human‐derived antibodies may offer translational advantages over previously reported camelid VHH‐based binders, particularly with respect to clinical development and immunogenicity considerations.

In contrast, RDS045 defines a structurally distinct mode of Stx1a recognition. Rather than approaching the canonical Gb3‐binding surface, RDS045 engages a non‐canonical lateral quaternary epitope formed across two adjacent Stx1aB protomers, thereby creating an inter‐protomer clamp on the assembled pentamer. This mode of recognition clearly differs from the receptor‐facing paradigm established by previously published anti‐Stx VHHs and expands the known structural landscape of Stx neutralization. The RDS045 interface is stabilized by an extensive hydrogen‐bonding network contributed by heavy chain CDR3 and light chain CDR1 across the protomer‐protomer boundary, as well as by insertion of LC‐CDR3 residue W94 into a lateral pocket on protomer A, where it forms a cation‐π interaction with R69 and additional local contacts that likely reinforce the bound state. Together with the proximity of the Fab to the Stx1aA region in the holotoxin model, these features support a mechanism that is not primarily explained by direct competition with Gb3, but rather by steric constraint on holotoxin architecture and/or interference with toxin structural integrity. Importantly, conservation mapping and sequence alignment showed that the RDS045 footprint contains several residues that are preserved among subtypes, raising the possibility that this quaternary surface represents a structurally constrained site of vulnerability. However, because RDS045 recognized a quaternary epitope formed across adjacent B‐subunit protomers, its conservation and recognition may also depend on interprotomer geometry and therefore require experimental validation in additional Stx subtypes. Considered together, RDS045 and RDS059 define two mechanistically distinct solutions for Stx1a neutralization, as one receptor‐proximal and canonical‐like, the other lateral, quaternary, and non‐canonical.

More broadly, our study highlights the value of human single‐B‐cell‐based antibody discovery for anti‐toxin therapeutic development. By screening antigen‐specific human memory B cells, we isolated fully human monoclonal antibodies with potent neutralizing activity, favorable binding properties, and, importantly, divergent structural mechanisms of action. This is a key conceptual advance of the present work. Previously reported structurally characterized anti‐Stx binders have largely come from camelid VHH or nanobody approaches, whereas our study shows that a human single‐B‐cell platform can recover not only potent neutralizers, but also mechanistically complementary antibodies directed to distinct epitopes on the same toxin target. That diversity has practical implications. Mechanism‐diverse antibodies such as RDS045 and RDS059 may provide a rational starting point for next‐generation anti‐Stx inhibitors, including antibody engineering, epitope‐guided optimization, and cocktail‐based therapeutic strategies designed to increase neutralization breadth, reinforce protection, and reduce the likelihood of functional escape. In this sense, the atomic‐resolution structures reported here do more than explain how two antibodies bind Stx1aB and also provide a structural framework for the future development of human anti‐Stx therapeutics with complementary mechanisms of action.

Although RDS045 and RDS059 neutralize Stx1a through distinct mechanisms, their partially overlapping epitopes and steric hindrance when bound to a single Stx1aB pentamer led to incomplete competition, as demonstrated by the BLI assay. Consequently, simply combining the two antibodies at high doses may not provide the anticipated therapeutic benefit. Further studies are needed to evaluate the efficacy of combination treatment and to determine the optimal antibody ratio, dose, and administration regimen.

Despite these advances, several limitations of the present study should be acknowledged. First, our analyses were centered on Stx1a, and it remains unclear to what extent the epitopes and neutralization mechanisms defined here extend to other clinically relevant Shiga toxin subtypes, particularly Stx2 variants that are more frequently associated with severe disease. Second, although our cell‐based and in vivo experiments support the protective activity of RDS045 and RDS059, these systems do not fully recapitulate the temporal and pathological complexity of human STEC infection and HUS, including tissue‐specific injury, systemic toxin trafficking, and host inflammatory responses. Third, the therapeutic window for effective antibody intervention was not comprehensively defined, and the relative contribution of neutralizing free versus cell‐associated or vesicle‐associated toxin remains unresolved. Fourth, only four of the 68 paired antibodies were confirmed to bind Stx1aB in ELISA, likely owing to the low abundance of Stx‐specific memory B cells in the donor, the deliberately inclusive sorting strategy, and cumulative losses during single‐cell antibody recovery. Finally, while the fully human origin of these antibodies supports their translational potential, additional studies will be required to assess pharmacokinetics, safety, developability, and the extent to which combining mechanistically distinct antibodies may improve breadth and robustness of protection. These limitations do not alter our central conclusion that human‐derived antibodies can neutralize Stx1a through distinct structural mechanisms, but they delineate the key experimental and translational questions that should be addressed in future studies.

## 4 Method Details

### 4.1 Gene Construction, Expression, and Purification of Shiga Toxin 1a B Subunits

Genes encoding Stx1aB of *E. coli* O157:H7 with C‐terminal AVI and His tags were synthesized de novo. Single amino acid alanine‐scanning mutagenesis of the fifth loop of Stx1aB (KTNACHNGGGFS) was performed to identify key binding residues. Ten mutant gene constructs of Stx1aB were generated by PCR using a site‐directed mutagenesis kit (TransGen Biotech), cloned into the pET‐SUMO(+) vector and transformed into *E. coli* BL21 strains (TransGen Biotech) for production of mutant Stx1aB proteins. Transformants were cultured overnight at 37°C in kanamycin‐supplemented LB medium, and were induced for protein expression at 18°C by addition of 0.2 mm IPTG. Cells were lysed by high‐pressure homogenization and used for purification of His‐tagged recombinant proteins by using Ni‐Charged MagBeads (GenScript). Purified recombinant proteins were stored at −80°C.

### 4.2 ELISA for Serum and Recombinant Antibody Titer

96‐well ELISA plates were coated with purified rStx1aB protein (200 ng/100 µL) in carbonate‐bicarbonate coating buffer (CBC, pH 9.6) and incubated overnight at 4°C. Plates were blocked with 300 µL/well of PBST containing 10% goat serum for 2 h at 37°C. Plasma samples at starting dilution of 1:50, and recombinant antibodies at starting concentration of 10 µg/mL were subjected to 3‐fold serial dilutions. An aliquot of 100 µL of each dilution was added per well and incubated for 1 h at 37°C. After washing with PBST, 100 µL/well of horseradish peroxidase (HRP)‐conjugated goat anti‐human IgG secondary antibody (1:10,000 dilution) was added and incubated for 1 h at 37°C. Plates were washed again, and 100 µL/well of 3,3',5,5'‐tetramethylbenzidine (TMB) substrate were added. The colorimetric reaction was allowed to proceed in the dark at 37°C for 5–10 min before being terminated with 2 m sulfuric acid. Absorbance was measured at 450 nm with a reference wavelength of 630 nm. Curve fitting and half‐maximal effective concentration (EC50) analysis were performed using GraphPad Prism software.

### 4.3 Isolation of Peripheral Blood Mononuclear Cells (PBMCs) and FACS Sorting of Single Memory B Cells

Venous blood from volunteers was collected in EDTA‐anticoagulant tubes. Plasma and PBMCs were isolated by Ficoll density gradient centrifugation. All procedures involving human samples were performed following protocols approved by the Ethics Committee of the Fifth Affiliated Hospital of Sun Yat‐sen University. Single memory B cells were sorted from PBMC by flow cytometry. Cells were stained with the cell surface lineage markers CD3, CD14, CD16, CD235a, CD20, CD27 for identifying memory B cells, and dual fluorescent dye‐labeled (PE‐Cy7 and BV421) rStx1aB for identifying Stx1aB antigen‐specific memory B cells. Data were analyzed using FlowJo software.

### 4.4 Single‐Cell Amplification of Human Immunoglobulin Variable Region Genes From B Cells

cDNAs were synthesized from the sorted single antigen‐specific memory B cells using Superscript *I–V* reverse transcriptase (Invitrogen) and gene‐specific primers, with incubation at 37°C for 1 h. VH and Vĸ/Vλ genes were amplified via nested PCR and sequenced. VH and VK/VL gene PCR products were used to assemble the full‐length linear Ig gene expression cassettes by overlap extension PCR. Linear cassettes encoding the full‐length immunoglobulin (Ig) heavy and light chains from a single B cell were used to co‐transfect HEK293T cells. After incubation for 6–8 h, the transfection medium was replaced with RPMI‐1640 medium containing 2% FBS (Life Technologies). Cells were cultured for 96 h at 37°C under 5% CO_2_, and culture supernatants were harvested for ELISA screening of antigen‐specific monoclonal antibodies.

### 4.5 Production of Recombinant Monoclonal Antibodies

Full‐length Ig heavy and light chain genes of the identified Stx1aB‐specific antibodies were cloned into the pcDNA3.1(+) mammalian expression vector for production of purified antibodies. Expi293 cells were transfected using polyethylenimine (Sigma–Aldrich). Culture supernatants were collected from the transfected culture after 4 days, and recombinant antibodies were purified using Protein A beads (GenScript). Purified antibodies were further characterized.

### 4.6 Surface Plasmon Resonance (SPR) for Binding Kinetics

Binding kinetics were assessed using a Biacore 8K system. Individual monoclonal anti‐Stx1aB antibodies were captured by anti‐human IgG (Fc) antibody immobilized on a sensor chip. rStx1aB protein at various concentrations was flowed over the sensor chip at 30 µL/min. A binding phase of 90 s and dissociation phase of 300 s were recorded. The sensor surface was regenerated by washing using 3 m MgCl_2_. Affinity constants were calculated using the Biacore 8K Evaluation Software.

### 4.7 VeroE6 Cell Viability Assay

VeroE6 cells were seeded at 5,000 cells/well and cultured for 3–5 h at 37°C. For neutralization assays, Stx1a toxin at its CC80 concentration (1 µg/mL) was pre‐incubated with 3‐fold serial dilutions of anti‐Stx1a antibodies at the starting concentration of 100 µg/mL before addition to cells. After 48 h of incubation, viability of cells was assessed using the CCK‐8 reagent. Absorbance at 450 nm was measured 1–4 h after addition of CCK‐8 reagent. Dose‐response curves were plotted using GraphPad Prism software.

### 4.8 Cryo‐EM Data Collection and Image Processing

Cryo‐EM data for Complex of RDS045/RDS059 Fab with rStx1aB were collected on a 300 kV Titan Krios G4 equipped with a Falcon 4i direct‐electron detector and a Selectris X energy filter (operating at 10 e^−^V slit). Movies were recorded in AFIS mode at 165 000× nominal magnification (0.738 Å pixel) with −1.0 to −2.0 µm defocus and a total dose of ∼55 e^−^/Å^2^. EPU software controlled automated acquisition.

Processing was performed in cryoSPARC [31]. After patch motion correction and CTF estimation, images were screened for defocus, CTF‐fit resolution, and ice thickness. Particles were picked with blob and template pickers, extracted, and subjected to iterative 2‐D classification. Selected particles were used for ab‐initio reconstruction and heterogeneous refinement. The best class was refined with C5 symmetry, followed by non‐uniform refinement, CTF refinement, and reference‐based motion correction, yielding a pentamer map at 2.12 Å high‐resolution for RDS045 Fab with rStx1aB and 2.00 Å high‐resolution for RDS059 Fab with rStx1aB.

To enhance interface details, the final particle set was symmetry‐expanded, and focused 3‐D classification was carried out with a monomer mask (one Fab + one/two antigen). The resulting class was locally refined, producing improved density for the interaction region. Local resolution was estimated within cryoSPARC, and the maps were sharpened with EMReady [32] prior to model building. A complete processing workflow is provided in Supplementary Materials (Figures S4 and S5).

### 4.9 Model Building and Refinement

AlphaFold3 [33] was used to generate these initial models of antigen and Fab. Then one monomer (one antigen + one Fab) was docked into the locally refined map in UCSF Chimera [34], manually adjusted in WinCoot [35], and subjected to iterative real‐space refinement in PHENIX [36]. Five copies of the refined monomer model were then positioned into the pentamer density, followed by a final real‐space refinement cycle. Model geometry was validated with MolProbity, and all figures were rendered in UCSF Chimera and ChimeraX [37].

### 4.10 Ability of Antibody to Block Stx1aB Binding to VeroE6 Cells Assed by Flow Cytometry

VeroE6 cells were detached by treatment with Versene solution (Gibco). Detached cells in suspension were incubated with wild‐type Stx1aB, Stx1aB mutant proteins, or Stx1aB pre‐mixed with antibody RDS059 on ice for 30 min. After washing, cells were stained with an anti‐His fluorescent antibody on ice for 30 min, washed again, and analyzed by flow cytometry. The mean fluorescence intensity (MFI) reflecting the binding of Stx1aB to VeroE6 cells was quantified using FlowJo software (v10.8.1).

### 4.11 Animal Infection and Antibody Treatment

Female BALB/c mice (6–8 weeks old) were randomly assigned to the indicated groups. For toxin challenge, mice were administered Stx1a holotoxin intraperitoneally at 2 × LD50 (0.1 mg/kg) on day 0. One hour after challenge, mice received a single intraperitoneal injection of either an irrelevant isotype control antibody (anti‐tetanus toxin antibody) or the indicated dose of RDS045 or RDS059. Unchallenged control mice received neither toxin nor antibody treatment.

For survival experiments, infected mice were treated with the indicated doses of RDS045 or RDS059, and survival was monitored daily for 21 days. Mice were observed for overt signs of morbidity, including reduced activity and ruffled fur.

All animal procedures were performed at Zhuhai Bestest Biotechnology Co., Ltd. and approved by its Institutional Animal Care and Use Committee (IACUC) (protocol no. IAC25W002), in accordance with institutional guidelines and applicable regulations.

### 4.12 Serum Biochemical Analysis and Histopathology

For serum biochemical and histopathological analyses, samples were collected from a predefined subset of mice. In the Stx1a‐only and isotype‐treated groups, blood and kidney samples were collected at the onset of overt clinical signs. In the unchallenged and antibody‐treated groups, samples were collected at day 21 if mice remained clinically well.

Blood samples were collected and serum was isolated by centrifugation. Urea concentrations were determined using the urease‐glutamate dehydrogenase assay, and blood urea nitrogen (BUN) was calculated using a conversion factor of 2.8. Serum creatinine was measured using the creatininase hydrolysis method. Serum total bilirubin was measured by the metavanadate oxidation method.

### 4.13 Biolayer Interferometry‐Based Competition Assay

The directional binding competition between RDS045 and RDS059 was evaluated using a sequential analyte‐binding assay on an Octet R8 system (Sartorius). All measurements were performed at room temperature (25°C) in PBST (0.02% Tween‐20). Streptavidin (SA) biosensors were first equilibrated in PBST to establish a baseline and were subsequently transferred to wells containing biotinylated recombinant Stx1aB (rStx1aB‐biotin, 5 µg/mL) for ligand immobilization. Antigen loading was continued until the BLI response reached approximately 50% of the maximum loading response. The antigen‐loaded biosensors were then transferred to wells containing the first analyte and incubated for 90 s. After the first analyte‐binding signal approached a plateau, the biosensors were transferred to wells containing the second analyte and incubated for 120 s. The biosensors were subsequently returned to PBST for dissociation.

To evaluate the ability of RDS045 to block RDS059 binding, the biosensors were sequentially exposed to RDS045 (600 nm) and a mixture containing RDS045 (600 nm) and RDS059 (300 nm). The corresponding RDS045→RDS045 condition was used as the self‐binding control, and the buffer→RDS059 condition was used to determine the maximum RDS059‐binding response. The reciprocal competition experiment was performed by sequentially exposing the biosensors to RDS059 (300 nm) and the RDS045/RDS059 mixture. The RDS059→RDS059 condition served as the self‐binding control, and the buffer→RDS045 condition was used to determine the maximum RDS045‐binding response. Signals were corrected by subtracting the response obtained from the corresponding reference biosensors and reference wells.

Sensorgrams were processed using Octet Analysis Studio. For each condition, the response during the second‐analyte step was quantified by averaging the BLI signal recorded during the final 10 s of the 120 s association period. The blocking percentage was calculated as shown in Figure S5. The blocking values were calculated independently for the two binding directions, namely RDS045→RDS059 and RDS059→RDS045. Measurements were performed in triplicate, and the results were expressed as the mean ± SD.

### 4.14 Statistical Analysis

All statistical analyses were performed using GraphPad Prism v10.6. For serum biochemical measurements, differences among groups were assessed by one‐way ANOVA followed by Dunnett's multiple‐comparisons test, using the Stx1aA plus isotype control or Stx1aA only group as the reference group. Data are presented as mean ± SD. A *p*‐value < 0.05 was considered statistically significant.

### 4.15 Quantification and Statistical Analysis

The statistical details for any given analyses are provided in the corresponding figure legend. Additional information about statistical analysis can be found in the relevant method details sections.

## Author Contributions

Conceptualization, S.C.; methodology, J.K., X.L., and X.W.; investigation, X.L., X.W., Z.H., S.X., Y.L., J.P., G.H. and Y.W.; Writing – original draft, S.C., X.L., X.W., and Y.W.; Writing – review and editing, S.C., and T.K., and C.W.; funding acquisition, S.C., and C.W.; resources, S.C., X.L., J.K., and Y.W.; supervision, S.C., J.K., C.W.

## Funding

This work is supported by the National Natural Science Foundation of China (92569303, 32561143026 to S. C.; 82470746 to W. C.), National Science & Technology Major Project of the Ministry of Science and Technology of China (2025ZD01903905 to S. C.), Sun Yat‐sen University supporting grant (2024_89000_B25886 to S.C.).

## Ethics Statement

All animal experimentation was approved by the Institutional Animal Care and Use Committee (IACUC) of Zhuhai Bestest Biotechnology Co., Ltd. (protocol no. IAC25W002), in accordance with institutional guidelines and applicable regulations.

## Consent

Written informed consent was obtained from all participants/patients involved in the study.

## Conflicts of Interest

Y.W. and X.W. are employees at Zhuhai Trinomab Biotechnology Co., Ltd. Antibodies (RDS045 and RDS059) are the subject of patent applications filed by Zhuhai Trinomab Biotechnology Co., Ltd. The remaining authors declare no competing interests.

## Supporting information




**Supporting File**: advs77402‐sup‐0001‐SuppMat.docx.

## Data Availability

The cryoEM density maps and corresponding atomic coordinates have been deposited in the Electron Microscopy Data Bank (EMDB) and Protein Data Bank (PDB), respectively. The accession codes are: EMD‐80320 and 25RG for RDS045‐Stx1aB, EMD‐80337 and 25SB for RDS059‐Stx1aB. The data that support the findings of this study are available from the corresponding author upon reasonable request.
