# Supplementary material for: Structural Basis of Shiga Toxin Neutralization by Human Antibodies Targeting Canonical Receptor‐Binding and Non‐Canonical Assembly Sites
Source: Adv Sci (Weinh). 2026 Aug 30:e77402. Online ahead of print. doi: 10.1002/advs.77402 (PMC13526710; doi:10.1002/advs.77402)
Supplement: Supplementary file 1 [file ADVS-9999-e77402-s001.docx]

**Structural Basis of Shiga Toxin Neutralization by Human Antibodies Targeting Canonical Receptor -Binding and Non-canonical Assembly Sites**

Jianting Ke^1#^, Xin Li^2#^, Xiaoli Wang^3#^, Zhaoxia Huang^2^, Shaojie Xue^2^, Yuyang Liu^2^, Jing Pan^2^, Guowei Hu^2^, Yayu Wang^3^*, Cheng Wang^1^* and Shoudeng Chen^2,4,5^*

^1^Division of Nephrology, Department of Medicine, The Fifth Affiliated Hospital, Sun Yat-sen University, Zhuhai, Guangdong, China

^2^Department of Experimental Research, The Fifth Affiliated Hospital, Sun Yat-sen University, Zhuhai, Guangdong, China

^3^Zhuhai Trinomab Pharmaceutical Co., Ltd, Zhuhai, Guangdong, China

^4^Guangdong Engineering Research Center of Nasopharyngeal Carcinoma Diagnosis and Therapy, Guangdong-Hong Kong-Macao University Joint Laboratory of Interventional Medicine, Guangdong Engineering Technology Research Center of Molecular Imaging, the Fifth Affiliated Hospital, Sun Yat-sen University, Zhuhai, Guangdong, China.

^5^State Key Laboratory of Anti-Infective Drug Discovery and Development, Sun Yat-sen University, Guangdong, China

^#^These authors contributed equally

*Corresponding Authors:

Shoudeng Chen (Lead contact), [chenshd5@mail.sysu.edu.cn](mailto:chenshd5@mail.sysu.edu.cn);

Cheng Wang, wangch2@mail.sysu.edu.cn;

Yayu Wang, wangyayu@trinomab.com

**Supplyment Information**

**Figure S1. Flow cytometric gating strategy for isolation of Stx1aB-bingding memory B cells from donor Ep040**

(A) Representative flow cytometry gating strategy used to identify and isolate Stx1aB-bingding memory B cells from PBMCs of donor Ep040. Sequential gates were applied to select singlets, and live cells, followed by exclusion of CD14^+^ monocytes and CD3^+^CD16^+^ cells. Memory B cells were defined as IgD^-/low^CD235a^-^CD27^+^ cells and were further analyzed for binding to fluorescently labeled Stx1aB. Stx1aB-bingding memory B cells were identified as double positive cells and sorted as single cells for antibody gene amplification.

(B) Numbers and frequencies of cells obtained at each gating step shown in (A). Absolute event counts, percentages of the parent population, and percentages of total events are summarized for each gate.

**Figure S2. Sequence features and functional characterization of antibodies recovered from Stx1aB-binding memory B cells.**

(A) Somatic mutation frequencies of the variable heavy (VH) and variable light (VK/VL) chains among 68 paired antibodies recovered from Stx1aB-binding memory B cells, shown as percentage divergence from germline sequences. Each dot represents one antibody sequence, and horizontal lines indicate the median.

(B) CDR3 lengths of the variable heavy (VH) and variable light (VK/VL) chains among the 68 paired antibodies recovered from Stx1aB-binding memory B cells. Each dot represents one antibody sequence, and horizontal lines indicate the median.

(C) ELISA binding curves of four candidate Stx1aB-binding antibodies, RDS005, RDS036, RDS045, and RDS059, against recombinant Stx1aB. Binding EC50 values are indicated.

(D) ELISA-based screening of 68 antibodies recovered from Stx1aB-binding memory B cells at about 0.5 μg/mL against recombinant Stx1aB. Binding signals are shown as OD450 values for individual antibodies.

(E) SPR analysis of RDS005 binding to recombinant Stx1aB. Representative sensorgrams obtained with the indicated analyte concentrations are shown. The calculated equilibrium dissociation constant (K*_D_*) is indicated.

(F) SPR analysis of RDS036 binding to recombinant Stx1aB. Representative sensorgrams obtained with the indicated analyte concentrations are shown. The calculated equilibrium dissociation constant (K*_D_*) is indicated.

(G) Dose-response curve of Stx1a holotoxin in VeroE6 cells, determined by CCK-8 assay. The percentage of dead cells is plotted against the indicated toxin concentrations, and the calculated CC50 and CC80 values are shown.

**Figure S3. Cryo-EM workflow for reconstruction of the Stx1aB-RDS059 Fab complex.**

(A) Purification and cryo-EM sample preparation of the Stx1aB-RDS059 Fab complex. Upper panel, size-exclusion chromatography profile of recombinant Stx1aB mixed with RDS059 Fab, with the peak corresponding to the assembled complex indicated for cryo-EM analysis. Middle panel, SDS-PAGE analysis of peak fractions confirming the presence of the purified complex. Lower panel, representative cryo-EM micrograph of the Stx1aB-RDS059 Fab complex.

(B) Cryo-EM image-processing workflow for reconstruction of the Stx1aB-RDS059 Fab complex. Selected micrographs were subjected to motion correction, particle picking, 2D classification, ab initio reconstruction, heterogeneous refinement, non-uniform refinement, CTF refinement, and polishing to generate the pentamer reconstruction. Symmetry expansion and masked 3D classification were subsequently performed to obtain the locally refined monomer map. Particle numbers and map resolutions at each stage are indicated.

(C) Gold-standard Fourier shell correlation (GSFSC) curves for the globally refined Stx1aB pentamer reconstruction, yielding a final resolution of 2.00 Å.

(D) Gold-standard Fourier shell correlation (GSFSC) curves for the locally refined monomer map obtained after masking, yielding a final resolution of 2.18 Å.

**Figure S4. Cryo-EM workflow for reconstruction of the Stx1aB-RDS045 Fab complex.**

(A) Purification and cryo-EM sample preparation of the Stx1aB-RDS045 Fab complex. Upper panel, size-exclusion chromatography profile of recombinant Stx1aB mixed with RDS045 Fab, with the peak corresponding to the assembled complex indicated for cryo-EM analysis. Middle panel, SDS-PAGE analysis of peak fractions confirming the presence of the purified complex. Lower panel, representative cryo-EM micrograph of the Stx1aB-RDS045 Fab complex.

(B) Cryo-EM image-processing workflow for reconstruction of the Stx1aB-RDS045 Fab complex. Selected micrographs were subjected to motion correction, particle picking, 2D classification, ab initio reconstruction, heterogeneous refinement, non-uniform refinement, CTF refinement, and polishing to generate the pentamer reconstruction. Symmetry expansion and masked 3D classification were subsequently performed to obtain the locally refined monomer map. Particle numbers and map resolutions at each stage are indicated.

(C) Gold-standard Fourier shell correlation (GSFSC) curves for the globally refined Stx1aB pentamer reconstruction, yielding a final resolution of 2.12 Å.

(D) Gold-standard Fourier shell correlation (GSFSC) curves for the locally refined monomer map obtained after masking, yielding a final resolution of 2.46 Å.

**Figure S5. BLI-based sequential competition assay for mapping the relative binding relationships between RDS045 and RDS059.**

(A) RDS045 was used as the first analyte, followed by a mixture of RDS045 and RDS059 as the second analyte. Sequential exposure to RDS045 followed by RDS045 was used as the self-binding control, whereas sequential exposure to buffer followed by RDS059 was used to determine the maximum RDS059-binding response. The blocking of RDS059 binding by RDS045 was calculated as 40.72 ± 4.532%.

(B) RDS059 was used as the first analyte, followed by a mixture of RDS045 and RDS059 as the second analyte. Sequential exposure to RDS059 followed by RDS059 was used as the self-binding control, whereas sequential exposure to buffer followed by RDS045 was used to determine the maximum RDS045-binding response. The blocking of RDS045 binding by RDS059 was calculated as 72.30 ± 5.378%.

**Figure S6. Sequence annotation and IMGT Perles representations of the variable domains of RDS045 and RDS059.**

(A) Amino acid sequences of the variable domains of RDS045 Fab. The heavy chain variable region is shown on top and the light chain variable region is shown below. CDR1, CDR2, and CDR3 are indicated.

(B) IMGT Perles representation of the RDS045 heavy-chain variable domain.

(C) IMGT Perles representation of the RDS045 light-chain variable domain.

(D) Amino acid sequences of the variable domains of RDS059 Fab. The heavy chain variable region is shown on top and the light chain variable region is shown below. CDR1, CDR2, and CDR3 are indicated.

(E) IMGT Perles representation of the RDS059 heavy-chain variable domain.

(F) IMGT Perles representation of the RDS059 light-chain variable domain.

**Figure S7. Structural and functional mapping of RDS045 and RDS059 epitopes on the Stx1aB pentamer..**

(A) Superposition of the RDS059-bound Stx1aB structure with the Gb3 analogue-bound Stx1aB structure (PDB: 1BOS). The Stx1aB pentamer is shown in light pink, and one protomer is highlighted in purple. The heavy and light chains of RDS059 Fab are shown in light blue and light green, respectively. The Gb3 analogue from the 1BOS structure is shown as black sticks. The superposition shows that RDS059 binds the receptor-facing surface of Stx1aB and spatially overlaps with the Gb3-binding region.

(B) Enlarged view of the boxed region in (A). CDR1-3 in heavy chain of RDS059 are positioned over the receptor-binding surface and sterically occlude the three reported Gb3-binding sites on Stx1aB.

(C) Flow cytometry analysis of additional loop 5 alanine-substitution mutants of Stx1aB. The mutants shown here correspond to loop 5 residues not included in Figure 3E. Binding of the indicated Stx1aB variants was assessed using the same flow cytometry assay as in Figure 3E, with RDS005 included as a control antibody.

(D) Surface representation of the Stx1aB pentamer showing the spatial relationship between the RDS045- and RDS059-binding sites. Residues contacted by RDS045 are shown in yellow, residues contacted by RDS059 are shown in light blue, overlapping residues are shown in forest green, and Gb3 binding residues are outlined in pink. Side and bottom views are shown.

(E) Structures of two previously reported nanobodies targeting Shiga toxin B subunits with experimentally determined complex structures. The Stx2aB-Nb113 complex (PDB: 6FE4) is shown at the top, with Stx2aB in gray and Nb113 in cyan. The Stx2eB-NbStx2e1 complex (PDB: 4P2C) is shown at the bottom, with Stx2eB in gray and NbStx2e1 in orange.

**Figure S8. Comparison of RDS045-bound and holotoxin-associated Stx1aB pentamers.**

(A) Structural superposition of the RDS045-bound Stx1aB pentamer with the holotoxin-associated Stx1aB pentamer from PDB 1DM0. The RDS045-bound and holotoxin-associated structures are shown in salmon and gray, respectively. The superposition was performed using the 345 equivalent Stx1aB residues and yielded a core RMSD of 0.8293 Å.

(B, C) Surface representations colored according to the B-factor distributions of the holotoxin-associated Stx1aB pentamer (PDB: 1DM0) (B) and the RDS045-bound Stx1aB pentamer (C). The color scales were defined independently for the two structures and are shown beside the corresponding surface representations.

(D) Residue-wise Cα positional deviations between the RDS045-bound and holotoxin-associated Stx1aB pentamers. The plot shows the mean positional deviation for each residue in the indicated protomer groups.

(E) Comparison of normalized residue-wise B factors between the RDS045-bound and holotoxin-associated Stx1aB pentamers.

**Figure S9. Conservation and subtype variation of the RDS045- and RDS059-contacting residues across Stx1 and Stx2 subtypes.**

(A) Conservation mapping of the Stx1aB pentamer based on ConSurf analysis using sequences from 19 Shiga toxin subtypes. Conserved residues are shown in gray and variable residues in purple. The RDS045 epitope is outlined in red. Side and bottom views are shown.

(B) Conservation mapping of the Stx1aB pentamer based on the same ConSurf analysis as in (A), with the RDS059 epitope outlined in green. Side and bottom views are shown.

(C) Multiple-sequence alignment of the corresponding Stx1 and Stx2 B-subunit regions containing the RDS045- and RDS059-contacting residues. The sequences were shaded according to the percentage identity among the aligned Stx subtypes, with darker blue indicating higher sequence identity. Residues contacted by RDS045 and RDS059 were marked by red and green outlines, respectively.

**Table S1. Characteristics of 68 paired heavy and light chain sequences from Ep040 Stx1aB-binding memory B cells.**

| Antibody | VH | DH | JH | H_mutated | Ig_Isotype | VK/L | JK/L | K/L_mutated | Ig_Isotype |
| --- | --- | --- | --- | --- | --- | --- | --- | --- | --- |
| RDS001 | IGHV3-30 | IGHD2-2 | IGHJ6 | 2.7% | IgG3 | IGKV1-5 | IGKJ2 | 5.4% | Kappa |
| RDS002 | IGHV3-20 | IGHD5-12 | IGHJ6 | 7.5% | IgG1 | IGKV2-28 | IGKJ4 | 23.5% | Kappa |
| RDS003 | IGHV4-39 | IGHD3-10 | IGHJ4 | 3.4% | IgM | IGKV1-5 | IGKJ1 | 3.6% | Kappa |
| RDS004 | IGHV4-34 | IGHD4-17 | IGHJ4 | 5.5% | IgG1 | IGKV2-28 | IGKJ3 | 3.7% | Kappa |
| RDS005 | IGHV4-59 | IGHD6-13 | IGHJ4 | 18.7% | IgG2 | IGKV1-39 | IGKJ4 | 16.8% | Kappa |
| RDS006 | IGHV1-3 | IGHD3-10 | IGHJ4 | 7.9% | IgG2 | IGKV3-20 | IGKJ2 | 8.5% | Kappa |
| RDS007 | IGHV3-7 | IGHD2-2 | IGHJ6 | 2.7% | IgG1 | IGKV1-39 | IGKJ1 | 5.0% | Kappa |
| RDS009 | IGHV1-3 | IGHD6-19 | IGHJ4 | 18.8% | IgG3 | IGKV3-15 | IGKJ1 | 12.9% | Kappa |
| RDS010 | IGHV4-39 | IGHD4-17 | IGHJ4 | 5.4% | IgG1 | IGKV3-15 | IGKJ2 | 3.9% | Kappa |
| RDS011 | IGHV7-4-1 | IGHD7-27 | IGHJ5 | 7.9% | IgG2 | IGKV4-1 | IGKJ1 | 8.7% | Kappa |
| RDS012 | IGHV3-74 | IGHD1-26 | IGHJ4 | 3.8% | IgA1 | IGKV3-20 | IGKJ3 | 5.8% | Kappa |
| RDS013 | IGHV3-53 | IGHD6-13 | IGHJ4 | 7.3% | IgG1 | IGKV3-15 | IGKJ2 | 3.2% | Kappa |
| RDS014 | IGHV1-3 | IGHD6-19 | IGHJ6 | 11.9% | IgG3 | IGKV3-15 | IGKJ4 | 10.7% | Kappa |
| RDS015 | IGHV5-51 | IGHD5-18 | IGHJ4 | 4.1% | IgG1 | IGKV4-1 | IGKJ2 | 3.0% | Kappa |
| RDS016 | IGHV3-11 | IGHD2-2 | IGHJ4 | 5.8% | IgA1 | IGKV3-15 | IGKJ4 | 5.0% | Kappa |
| RDS017 | IGHV3-53 | IGHD1-26 | IGHJ4 | 6.2% | IgA1 | IGKV3-15 | IGKJ2 | 7.2% | Kappa |
| RDS018 | IGHV5-51 | IGHD1-1 | IGHJ2 | 4.8% | IgM | IGKV4-1 | IGKJ2 | 4.0% | Kappa |
| RDS019 | IGHV1-69 | IGHD2-21 | IGHJ3 | 6.6% | IgG1 | IGKV3-15 | IGKJ1 | 2.5% | Kappa |
| RDS020 | IGHV4-39 | IGHD1-26 | IGHJ4 | 5.4% | IgA1 | IGKV4-1 | IGKJ4 | 8.6% | Kappa |
| RDS021 | IGHV1-69 | IGHD5-24 | IGHJ4 | 4.5% | IgG1 | IGKV1-16 | IGKJ4 | 4.6% | Kappa |
| RDS022 | IGHV3-23 | IGHD4-17 | IGHJ4 | 5.6% | IgA1 | IGKV3-11 | IGKJ4 | 3.6% | Kappa |
| RDS023 | IGHV3-53 | IGHD1-26 | IGHJ4 | 5.6% | IgA1 | IGKV3-15 | IGKJ2 | 6.9% | Kappa |
| RDS024 | IGHV3-23 | IGHD5-24 | IGHJ4 | 6.5% | IgM | IGKV4-1 | IGKJ2 | 6.5% | Kappa |
| RDS025 | IGHV3-33 | IGHD3-16 | IGHJ4 | 2.4% | IgG3 | IGKV3-15 | IGKJ1 | 3.2% | Kappa |
| RDS026 | IGHV1-46 | IGHD3-16 | IGHJ4 | 3.4% | IgG1 | IGKV1-39 | IGKJ1 | 3.6% | Kappa |
| RDS028 | IGHV3-30 | IGHD2-15 | IGHJ4 | 8.0% | IgG2 | IGKV3-20 | IGKJ1 | 5.0% | Kappa |
| RDS029 | IGHV1-69 | IGHD3-22 | IGHJ6 | 1.4% | IgM | IGKV4-1 | IGKJ1 | 3.7% | Kappa |
| RDS030 | IGHV1-3 | IGHD6-19 | IGHJ2 | 2.1% | IgG1 | IGKV1-5 | IGKJ1 | 3.6% | Kappa |
| RDS031 | IGHV3-30 | IGHD6-19 | IGHJ6 | 4.6% | IgG3 | IGKV2-24 | IGKJ4 | 5.2% | Kappa |
| RDS032 | IGHV4-4 | IGHD3-10 | IGHJ6 | 6.9% | IgG1 | IGKV3-20 | IGKJ1 | 7.0% | Kappa |
| RDS033 | IGHV4-31 | IGHD3-16 | IGHJ4 | 21.7% | IgA1 | IGKV1-5 | IGKJ1 | 10.9% | Kappa |
| RDS034 | IGHV1-3 | IGHD2-2 | IGHJ4 | 13.8% | IgG1 | IGKV1-5 | IGKJ3 | 10.9% | Kappa |
| RDS035 | IGHV3-23 | IGHD2-15 | IGHJ4 | 15.8% | IgG3 | IGKV1-39 | IGKJ1 | 14.7% | Kappa |
| RDS036 | IGHV1-2 | IGHD6-19 | IGHJ4 | 5.8% | IgG1 | IGKV3-11 | IGKJ2 | 4.7% | Kappa |
| RDS037 | IGHV1-3 | IGHD1-26 | IGHJ3 | 6.6% | IgG1 | IGKV1-9 | IGKJ2 | 6.5% | Kappa |
| RDS038 | IGHV4-4 | IGHD3-10 | IGHJ4 | 3.1% | IgA1 | IGKV1-27 | IGKJ1 | 3.6% | Kappa |
| RDS039 | IGHV4-39 | IGHD6-19 | IGHJ6 | 8.8% | IgG1 | IGKV3-20 | IGKJ2 | 5.6% | Kappa |
| RDS040 | IGHV4-39 | IGHD2-8 | IGHJ6 | 13.0% | IgG2 | IGKV2-28 | IGKJ4 | 7.1% | Kappa |
| RDS041 | IGHV3-23 | IGHD3-10 | IGHJ4 | 3.8% | IgM | IGKV1D-12 | IGKJ4 | 7.6% | Kappa |
| RDS042 | IGHV1-2 | IGHD4-17 | IGHJ5 | 15.0% | IgG3 | IGKV1-5 | IGKJ1 | 12.3% | Kappa |
| RDS043 | IGHV3-23 | IGHD3-10 | IGHJ4 | 9.6% | IgG2 | IGKV3-20 | IGKJ5 | 14.4% | Kappa |
| RDS044 | IGHV1-46 | IGHD3-10 | IGHJ4 | 6.9% | IgA1 | IGKV1-9 | IGKJ2 | 6.1% | Kappa |
| RDS045 | IGHV1-2 | IGHD1-14 | IGHJ4 | 9.9% | IgG1 | IGKV3-11 | IGKJ2 | 7.5% | Kappa |
| RDS046 | IGHV3-74 | IGHD6-19 | IGHJ4 | 10.3% | IgA1 | IGKV1-39 | IGKJ1 | 3.6% | Kappa |
| RDS047 | IGHV4-31 | IGHD6-19 | IGHJ4 | 8.5% | IgG3 | IGKV1-16 | IGKJ4 | 8.2% | Kappa |
| RDS048 | IGHV3-74 | IGHD1-26 | IGHJ4 | 6.2% | IgG2 | IGLV3-16 | IGLJ2 | 8.9% | Lambda |
| RDS049 | IGHV4-39 | IGHD5-18 | IGHJ4 | 5.1% | IgA1 | IGLV2-14 | IGLJ3 | 8.2% | Lambda |
| RDS050 | IGHV1-3 | IGHD6-25 | IGHJ3 | 7.2% | IgG1 | IGLV3-1 | IGLJ3 | 6.1% | Lambda |
| RDS052 | IGHV4-59 | - | IGHJ6 | 3.8% | IgG3 | IGLV4-3 | IGLJ3 | 3.0% | Lambda |
| RDS054 | IGHV3-11 | IGHD6-19 | IGHJ4 | 1.7% | IgA1 | IGLV5-45 | IGLJ3 | 1.3% | Lambda |
| RDS059 | IGHV4-39 | IGHD3-16 | IGHJ5 | 11.8% | IgA1 | IGLV2-8 | IGLJ2 | 3.5% | Lambda |
| RDS060 | IGHV3-7 | IGHD2-21 | IGHJ4 | 3.4% | IgA1 | IGLV2-14 | IGLJ2 | 1.7% | Lambda |
| RDS061 | IGHV1-2 | IGHD6-13 | IGHJ4 | 7.2% | IgA1 | IGLV1-44 | IGLJ2 | 3.8% | Lambda |
| RDS062 | IGHV4-39 | IGHD5-24 | IGHJ4 | 5.1% | IgG1 | IGLV1-51 | IGLJ3 | 5.1% | Lambda |
| RDS063 | IGHV3-74 | IGHD6-19 | IGHJ4 | 10.9% | IgA1 | IGLV2-14 | IGLJ3 | 6.5% | Lambda |
| RDS064 | IGHV3-74 | IGHD6-19 | IGHJ4 | 10.6% | IgA1 | IGLV2-14 | IGLJ3 | 5.2% | Lambda |
| RDS065 | IGHV3-74 | IGHD6-19 | IGHJ4 | 9.9% | IgM | IGLV2-14 | IGLJ3 | 4.5% | Lambda |
| RDS067 | IGHV1-2 | IGHD1-14 | IGHJ6 | 1.7% | IgM | IGLV2-14 | IGLJ3 | 1.4% | Lambda |
| RDS068 | IGHV3-74 | IGHD6-19 | IGHJ4 | 10.9% | IgA1 | IGLV2-14 | IGLJ3 | 4.5% | Lambda |
| RDS069 | IGHV4-61 | IGHD1-1 | IGHJ4 | 13.8% | IgA1 | IGLV5-45 | IGLJ3 | 7.2% | Lambda |
| RDS071 | IGHV4-59 | IGHD6-19 | IGHJ4 | 11.0% | IgM | IGLV2-14 | IGLJ3 | 6.9% | Lambda |
| RDS072 | IGHV5-51 | IGHD5-12 | IGHJ4 | 8.9% | IgG3 | IGLV1-40 | IGLJ3 | 8.4% | Lambda |
| RDS073 | IGHV3-49 | IGHD2-15 | IGHJ4 | 10.6% | IgG3 | IGLV1-51 | IGLJ3 | 11.0% | Lambda |
| RDS074 | IGHV3-74 | IGHD6-19 | IGHJ4 | 9.2% | IgA1 | IGLV2-14 | IGLJ3 | 3.8% | Lambda |
| RDS075 | IGHV3-74 | IGHD6-19 | IGHJ4 | 9.2% | IgA1 | IGLV2-14 | IGLJ3 | 3.8% | Lambda |
| RDS077 | IGHV3-74 | IGHD6-19 | IGHJ4 | 10.2% | IgA1 | IGLV2-14 | IGLJ3 | 5.9% | Lambda |
| RDS078 | IGHV3-74 | IGHD6-19 | IGHJ4 | 10.9% | IgM | IGLV2-14 | IGLJ3 | 3.8% | Lambda |
| RDS079 | IGHV3-74 | IGHD6-19 | IGHJ4 | 8.5% | IgG2 | IGLV2-14 | IGLJ3 | 4.5% | Lambda |

**Table S2. SPR binding kinetics of RDS005, RDS036, RDS045, and RDS059 for Stx1aB.**

| Antibody | Ka(1/Ms) | kd (1/s) | KD (M) |
| --- | --- | --- | --- |
| RDS005 | 1.43×10^4^ | 4.97×10^-4^ | 3.48×10^-8^ |
| RDS036 | 5.99×10^4^ | 4.45×10^-4^ | 7.43×10^-9^ |
| RDS045 | 4.75×10^4^ | 4.16×10^-4^ | 8.76×10^-9^ |
| RDS059 | 6.01×10^4^ | 5.35×10^-4^ | 8.89×10^-9^ |

**Table S3.** **Cryo-EM data collection, refinement and validation statistics, Related to Figures 2-4 and Figures S4-5.**

|  | RDS045:Stx1aB  (PDB:25RG;EMDB:EMD-80320) | RDS059:Stx1aB  (PDB:25SB; EMDB: EMD-80337) |
| --- | --- | --- |
| Data collection and processing |  |  |
| Magnification | 165,000 | 165,000 |
| Voltage (kV) | 300 | 300 |
| Defocus range (μm) | -1.0~-2.0 | -1.0~-2.0 |
| Pixel size (Å) | 0.738 | 0.738 |
| Symmetry imposed | C5 | C5 |
| Initial particle images (no.) | 21456 | 13614 |
| Final particle images (no.) | 18806 | 13432 |
| Map resolution (Å) | 2.12 | 2.00 |
| FSC threshold | 0.143 | 0.143 |
| Refinement |  |  |
| Initial model used (PDB code) | N/A | N/A |
| Model resolution (Å) | 2.42 | 2.35 |
| FSC threshold | 0.5 | 0.5 |
| Model resolution range (Å) | 2.06~12.0 | 1.95~12.0 |
| Map sharpening B factor (Å2) | 76.6 | 61.4 |
| Model composition |  |  |
| Non-hydrogen atoms | 19365 | 19240 |
| Protein residues | 2545 | 2560 |
| B factors (Å2) |  |  |
| Protein | 103.14 | 44.87 |
| R.m.s. deviations |  |  |
| Bond lengths (Å) | 0.004 | 0.005 |
| Bond angles (°) | 0.595 | 0.671 |
| Validation |  |  |
| MolProbity score | 1.75 | 1.47 |
| Clashscore | 4.32 | 2.93 |
| Poor rotamers (%) | 3.79 | 1.78 |
| Ramachandran plot |  |  |
| Favored (%) | 97.53 | 96.8 |
| Allowed (%) | 2.47 | 3 |
| Disallowed (%) | 0 | 0.2 |
